# Function Analysis of Cholesterol 7-Desaturase in Ovarian Maturation and Molting in *Macrobrachium nipponense*: Providing Evidence for Reproductive Molting Progress

**DOI:** 10.3390/ijms24086940

**Published:** 2023-04-08

**Authors:** Jisheng Wang, Sufei Jiang, Wenyi Zhang, Yiwei Xiong, Shubo Jin, Dan Cheng, Yalu Zheng, Hui Qiao, Hongtuo Fu

**Affiliations:** 1 Wuxi Fisheries College, Nanjing Agricultural University, Wuxi 214081, China; 2 Key Laboratory of Freshwater Fisheries and Germplasm Resources Utilization, Ministry of Agriculture and Rural Affairs, Freshwater Fisheries Research Center, Chinese Academy of Fishery Sciences, Wuxi 214081, China

**Keywords:** *Macrobrachium nipponense*, Cholesterol 7-desaturase, molting, ovarian maturation

## Abstract

The Cholesterol 7-desaturase gene plays an important role in insect ecdysone synthesis, but its role in ovarian development has not been reported. In this study, characteristics and the phylogenetic relationship of Cholesterol 7-desaturase were identified by bioinformatics. qPCR showed that the *Mn-CH7D* gene was highly expressed in the ovary, which was much higher than that in other tissues, and the expression level of *Mn-CH7D* reached the highest level at the third stage of the ovarian development stage (O-III). During embryonic development, the *Mn-CH7D* gene expression was highest in the zoea stage. The function of the *Mn-CH7D* gene was explored by RNA interference. The experimental group was injected with *Mn-CH7D* dsRNA through the pericardial cavity of *M. nipponense*, while the control group was injected with the same volume of *dsGFP*. Statistical analysis of gonadal development and GSI calculation showed that the silencing of *Mn*-*CH7D* resulted in the suppression of gonadal development. In addition, the molting frequency of the experimental group was significantly lower than that of the control group during the second molting cycle after silencing *Mn-CH7D*. On the seventh day after silencing, ecdysone content in the experimental group was significantly reduced. These results demonstrated that the *Mn-CH7D* gene played a dual role in ovarian maturation and molting of *M. nipponense*.

## 1. Introduction

*Macrobrachium nipponense* (Class: Crustacea, Order: Decapoda) is an economically important freshwater aquaculture species in China and the only indigenous species in large-scale aquaculture freshwater shrimp [1]. This species has the advantages of a short breeding cycle, small investment, less disease, strong adaptability, stable price, and so on [2]. In large-scale breeding, it was found that after the female prawns entered the breeding period (April to October), especially when the water temperature increased to more than 22 °C, the gonadal maturation cycle was greatly shortened, and the offspring were multiplied in large numbers in the pond, resulting in multi-generation reunion, high breeding density, large feed consumption, and large risk of hypoxia [3,4]. At the same time, the growth of the original female shrimp was affected, leading to a general miniaturization, and the market size of shrimp was greatly reduced; this phenomenon of rapid sexual maturation is very prevalent in shrimp [5,6]. It seriously affects the production and economic benefit of shrimp farming [7]. In response to this problem, the research team screened many genes related to rapid sexual maturity [8,9]. In the hepatopancreas transcriptome of female *M. nipponense* adult ovaries from the O-I to O-V stages of development, KEGG enrichment revealed that the “insect hormone synthesis” signaling pathway was closely related to ovarian development. The Cholesterol 7-desaturase (*CH7D*) is significantly expressed in this pathway. Therefore, the role of cholesterol 7-desaturase in molt and ovarian maturation were further investigated.

Molting plays an extremely important role in the growth, development, reproduction, and survival of crustaceans and runs through their entire life cycle [10]. During the breeding period, female molting is interwoven with ovarian development, specifically manifested in the need to molt before holding eggs [11]. However, this phenomenon has rarely been confirmed, and the related genes have not been found. In crustaceans, ecdysis and gonadal development are two important physiological processes that are closely linked and interact with each other, both of which require a large amount of material energy and the regulation of endogenous hormone signaling pathways. During the breeding season, most of the female energy is adjusted for gonad development to meet the reproductive demand [12,13,14], so the energy mobilized for growth will reduce, resulting in slow growth, small specification, and failure to meet the market requirements, which affects production and economic benefit. Studies have shown that the levels of various ecdysones in insects were related to regulating physiological and developmental processes in the ecdysone synthesis pathway of insects [15,16]. Tiu S H K et al. [17] found that ecdysone could promote ovarian development, and similar results were found in *Acanthinyx lunulatus* [18] and *Penaeus vannamei* [19]. The biosynthesis and metabolic pathways of ecdysone in insects have been well studied [20,21,22]. However, the biosynthetic pathways and the enzymes involved in ecdysteroid biosynthesis in crustaceans remain less well understood. In addition, previous studies on ecdysone mainly focused on growth, development, and morphological regulation [23,24]. The mechanism of regulation and coordination between ecdysgenesis and gonadal development is poorly understood. Therefore, it is of great significance to study the correlation between molting and gonad maturation of female *M. nipponense*, both in terms of technology and production.

In this study, sequence characteristics and phylogenetic relationships of *Mn-CH7D* were analyzed by bioinformatics. The expression patterns of the *Mn-CH7D* gene in different tissues, different stages of the ovary, and different stages of embryo development of *M. nipponense* were investigated by qPCR. The localization of *Mn-CH7D* mRNA in the ovary was detected by in situ hybridization (ISH). In addition, the regulation of the *Mn-CH7D* gene on molting and ovarian maturation of *M. nipponense* was studied using RNA interference technology (RNAi). After the *Mn-CH7D* gene was knocked down by RNAi, the function of *Mn-CH7D* in the molting and ovarian maturation of *M. nipponense* was confirmed by observing the molting number and ovarian development. Finally, the content of ecdysone in *M. nipponense* was detected by ELISA. 

## 2. Results

### 2.1. Full-Length Sequence Analysis of Mn-CH7D

**Figure 1:**
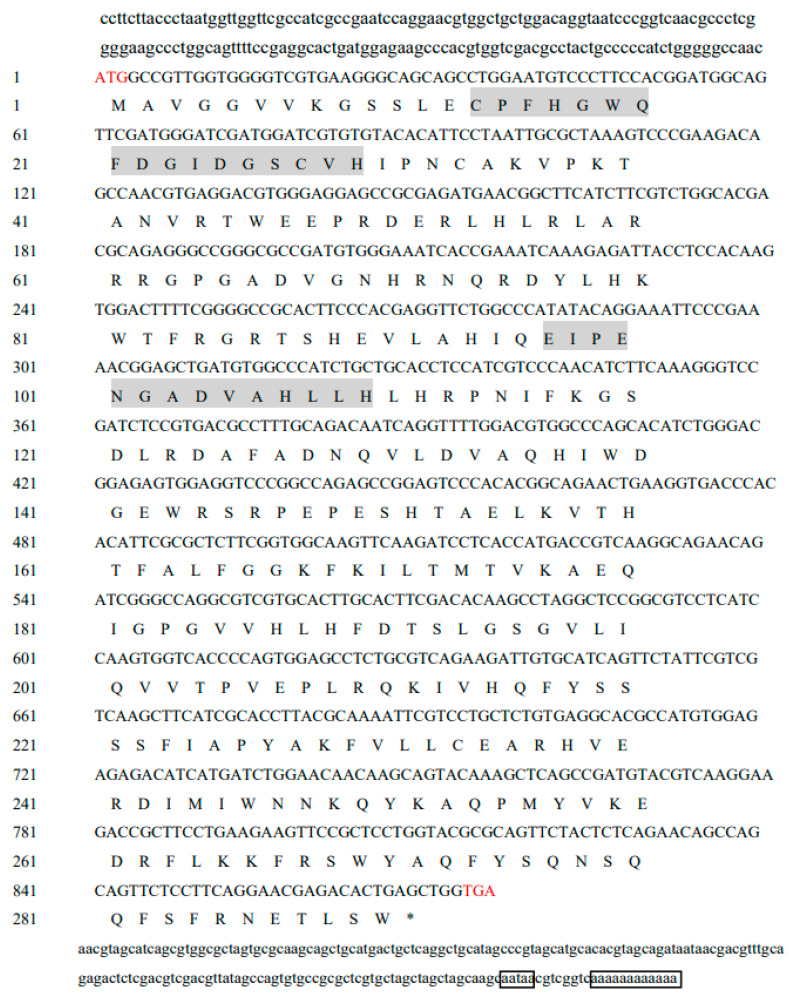
Full cDNA sequence and predicted amino acid sequence of *Mn-CH7D*. The start codon ATG and the stop codon TAG are marked with red. The gray shades are two conserved domains, in order: the Rieske [2Fe-2S] domain and the non-heme iron binding domain. The black box represents the tailing signal and poly(A). The stop codon TAG in the amino acid sequence is represented by an asterisk (*).

The full-length Mn-CH7D cDNA sequence determined by combining a subset of sequences from the *M. nipponense* hepatopancreas transcriptome library with the 3′race production was found to be 1225 bp (GenBank accession no.OQ553932). The open reading frame (ORF) was 876 bp long, encoding 291 amino acids, the 5′ non-coding region (5′UTR) contained 162 bp, and the 3′UTR contained 187 bp. The cDNA sequence and amino acid sequence of the Mn-CH7D gene are shown in Figure 1. According to the analysis, the molecular weight (Mw) of the Mn-CH7D protein was 33,550.12 Da, and the theoretical isoelectric point was 9.12. The predicted full-length amino acid composition of Mn-CH7D showed that the serine (Valine, V) content was the highest (7.9%) while the cysteine (Cys, C) and methionine (Met, M) content was the lowest (1.4%). Among all the amino acids, there were 37 positively charged amino acids (Arg + Lys) and 31 negatively charged amino acids (Asp + Glu). Sequence analysis showed that Mn-CH7D contained two conserved structure domains involved in enzyme catalysis: the Rieske [2Fe-2S] domain and the non-heme iron binding domain. In addition, the amino acid sequence of Mn-CH7D detected by Signal 5.0 analysis showed no signal peptide sequence.

### 2.2. Similarity Comparison and Phylogenetic Analysis

**Figure 2:**
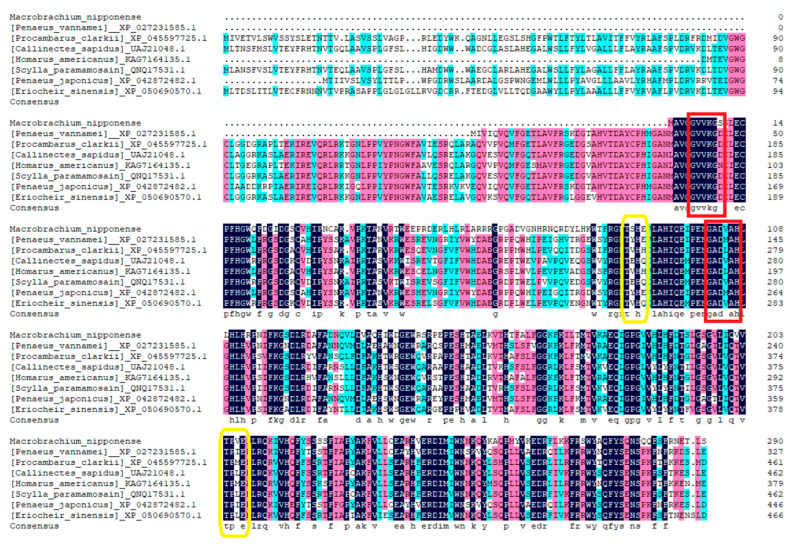
Alignment of the deduced amino acid sequence of *Mn-CH7D* with other species. N-myristoylation sites are marked by red boxes, and Casein kinase II phosphorylation sites are marked by yellow boxes.

Using the DNAMAN 6.0 software, the amino acid sequences of the *Mn-CH7D* gene were homologous to more than 10 species, with multiple sequence comparisons performed. The results showed the gene similarity between *Mn-CH7D* and *Penaeus vannamei*, *Procambarus clarkii*, *Callinectes sapidus*, *Homarus americanus*, *Scylla paramamosain*, *Penaeus japonicus*, and *Eriocheir sinensis* of 64.65%, 65.19%, 60.88%, 64.85%, 61.90%, 65.32%, and 60.46%, respectively (Figure 2).

**Figure 3:**
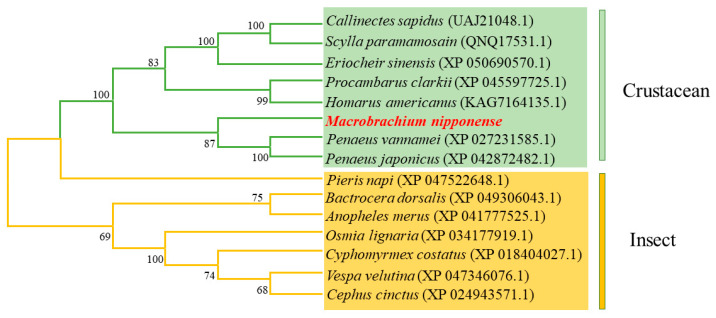
Phylogenetic tree connecting the *Mn-CH7D* amino acid sequence of *Macrobrachium nipponense* and other species. The numbers in brackets indicate the GenBank accession numbers. The numbers shown at the branches indicate the bootstrap values (%).

Using the MEGA 7.0 software, the amino acid sequence of the *Mn-CH7D* gene and the amino acid sequence of the *Mn-CH7D* gene of other species were used for phylogenetic analysis. Phylogenetic tree analysis showed that *Mn-CH7D* first clustered with *Penaeus vannamei*, *Penaeus japonicus*, *Procambarus clarkii*, and other crustaceans and then clustered with insects, such as *Pieris napi*, *Bactrocera dorsalis*, *Anopheles merus*, and others (Figure 3).

### 2.3. Tissue-Specific Gene Expression of Mn-CH7D

**Figure 4:**
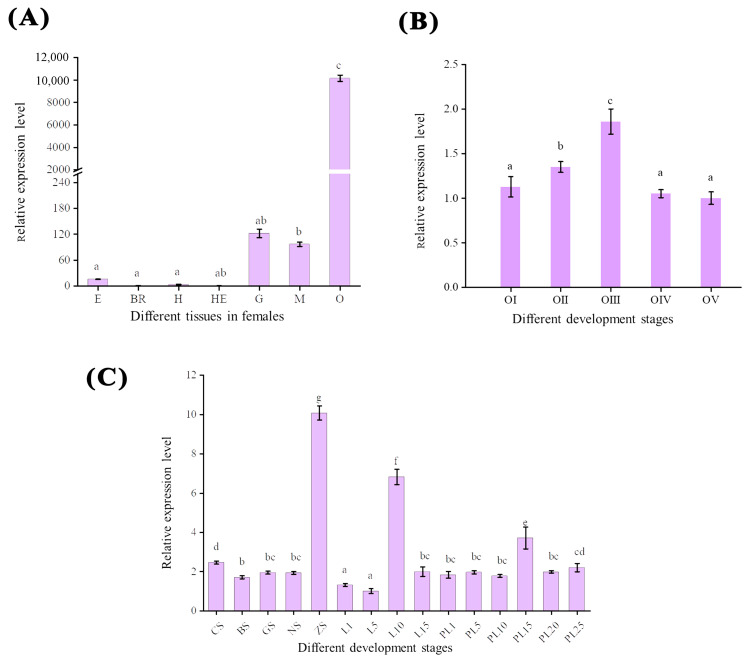
The expression pattern of the *Mn-CH7D* gene in different tissues (**A**), different stages of ovarian maturation (**B**), and developmental stages (**C**) of *Macrobrachium nipponense* were measured by qPCR. E: eyestalk, Br: cerebral ganglion, H: heart, He: hepatopancreas, G: gill, M: muscle, O: ovary; Data are presented as the mean ± SD (n = 6). Different letters indicate significant differences. *p* < 0.05 was considered to be statistically significant.

In the seven tissues of *M. nipponense*, the ovary had the highest expression, which was much higher than that in other tissues (*p <* 0.05). *Mn-CH7D* was weakly expressed in the muscle and gill. The relative expression levels of mRNA detected in the brain and hepatopancreas were the lowest (Figure 4A). In the ovary (Figure 4B), the expression level of *Mn-CH7D* increased significantly from stage O-I to stage O-III, reaching a maximum at the secondary vitellogenesis (O-III) stage (*p <* 0.05). The expression level of *Mn-CH7D* decreased sharply from stage O-III to stage O-IV, and there was no significant difference between O-IV and O-V or between O-V and O-I (*p >* 0.05). 

*Mn-CH7D* was expressed in both the embryonic and metamorphic developmental stages (Figure 4C). At the early embryonic development stage, the expression level of *Mn-CH7D* was higher at the cleavage stage (CS) than at the blastula stage (BS) (*p <* 0.05). Subsequently, as the embryo developed, the expression level of *Mn-CH7D* reached the highest level in the zoea stage (ZS) (*p <* 0.05). During larval development, the expression level of the *Mn-CH7D* gene was higher on the 10th day (L10) after hatching (*p <* 0.05). After metamorphosis, the expression level of the *Mn-CH7D* gene was the highest at PL15, while there was no significant difference in other developmental stages (*p >* 0.05).

### 2.4. Localization of Mn-CH7D at Different Stages of Ovarian Development

**Figure 5:**
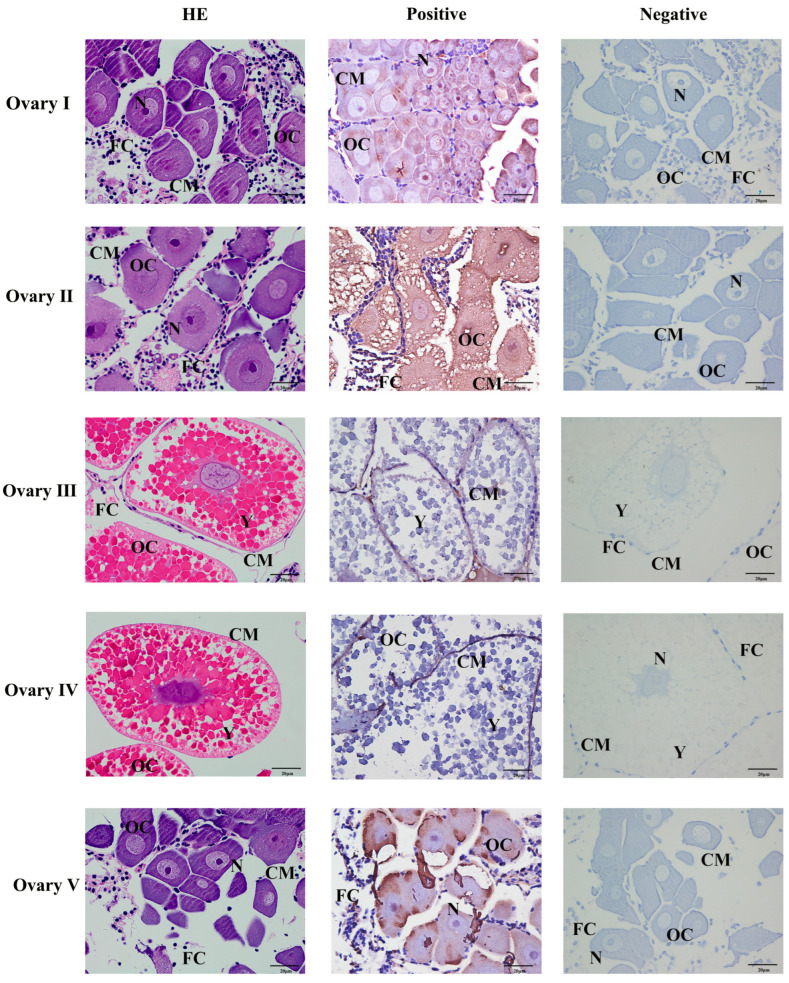
Localization of the expression of *Mn-CH7D* mRNA in *Macrobrachium nipponense* ovaries using in situ hybridization. OC: oocyte; N: nucleus; CM: cytoplasmic membrane; Y: yolk granule; FC: follicle cell; Scale bars: High magnification 400×.

The position of Mn-CH7D at different stages of ovarian development was located by ISH (Figure 5). The results showed obvious Mn-CH7D signals mainly distributed in intercellular space and cell membranes from the O-I stage to the O-IV stage. The signal of Mn-CH7D in the ovary was significantly enhanced during stage O-I to stage O-III and gradually weakened after stage O-IV. Moreover, the Mn-CH7D signal was detected in all five ovarian maturation stages, being strongest in O-III.

### 2.5. Functional Analysis of Mn-CH7D

#### 2.5.1. Interference Efficiency

**Figure 6:**
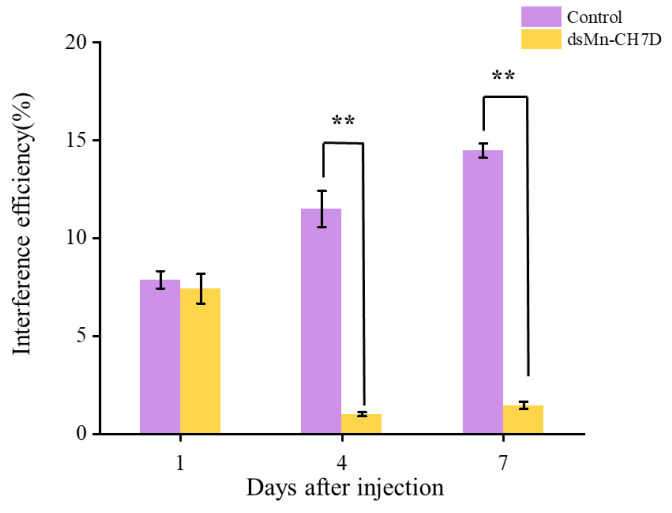
Expression levels of *Mn-CH7D* in ovaries of *Macrobrachium nipponense* after injection with *Mn-CH7D* dsRNA. Data are shown as mean ± SD (n = 6). “**” indicates the significance of the differences (*p* < 0.01).

To further explore the function of the *Mn-CH7D* gene in molting and ovarian maturation, RNAi technology was applied. The results in Figure 6 indicated that compared with the control group, the expression level of *Mn-CH7D* was down-regulated by 91.27% and 89.92% on days 4 and 7 after injection, respectively (*p <* 0.05).

#### 2.5.2. Effect of Mn-CH7D Knockdown on Ovarian Development of *M. nipponense*

**Figure 7:**
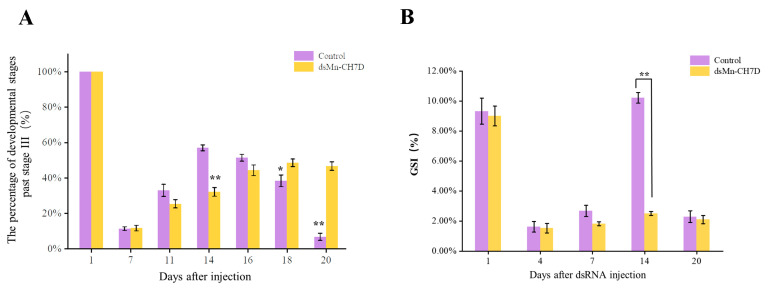
The percentage of development stages past stage III (**A**) and GSI (%) (**B**) of female *Macrobrachium nipponense* after injection with *Mn-CH7D* dsRNA. Data are shown as mean ± SD (n = 6). “*” indicates the significance of the differences (*p* < 0.05). “**” indicates the significance of the differences (*p <* 0.01).

The ovarian development of the experimental group and the control group is shown in Figure 7A. At the beginning of the experiment, shrimp in stage IV of ovarian development were selected. After 6 days, most of the ovaries of shrimp developed to stage II in both the experimental (85.72%) and control groups (96.74%). On the 14th day, the ovarian development in the control group reached stage IV (52.83%), while the experimental group remained in stage II (78.81%) (*p <* 0.05). After that, the percentage of developmental stages past stage III in the control group gradually declined. On the 16th day, part of the ovarian development in the control group entered the next round, while the experimental group remained in the previous round.

#### 2.5.3. Effect of Mn-CH7D Knockdown on Gonadal Development Index of *M. nipponense*

The gonadal development index (GSI) data were consistent with the ovarian development data (Figure 7B). On the first day of injection, there was no significant difference in GSI between the experimental group and the control group (*p >* 0.05). On day 14, there were significant differences in the GSI, 10.21% in the control group and 2.50% in the experimental group (*p <* 0.05). On day 20, the control group entered the next round of ovarian development, while the experimental group stagnated in stage II of the previous round.

#### 2.5.4. Effect of Mn-CH7D Knockdown on Molting Frequency of *M. nipponense*

**Figure 8:**
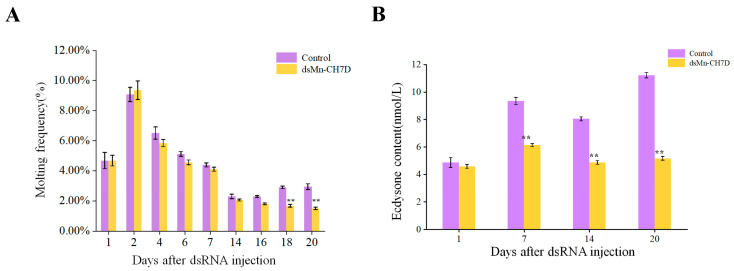
The molting frequency (**A**) and ecdysone content (**B**) of *Macrobrachium nipponense* after injection with *Mn-CH7D* dsRNA. Data are shown as mean ± SD (n = 6). “**” indicates the significance of the differences (*p <* 0.01).

Figure 8A shows the molting frequency of *M. nipponense* in the control and experimental groups after the *Mn-CH7D* knock. During the first round of development, from the first day to the seventh, molting frequency showed no differences between both the experimental group and the control group (*p >* 0.05). However, the control group of *M. nipponense* began the second round of concentrated molting on the 14th day. In contrast, there was almost no molting in the experimental group in the second week (*p <* 0.05).

#### 2.5.5. Effect of Mn-CH7D Knockdown on Ecdysis Hormone Content of *M. nipponense*

During the interference experiment, ecdysone content in *M. nipponense* was detected on day 1, day 7, day 14, and day 20 (Figure 8B). There was no significant difference between the control group and the experimental group on the first day after injection (*p >* 0.05). From the first day to the seventh day after injection, the content of ecdysone in both the experimental group and the control group increased and then decreased. From the seventh day, the ecdysone content of the experimental group was significantly lower than that of the control group (*p <* 0.05).

### 2.6. Tissue Section

**Figure 9:**
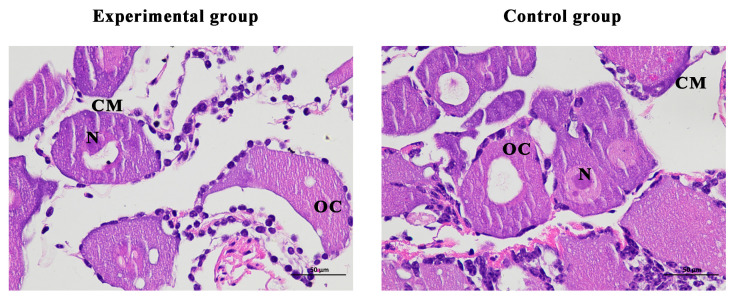
Histological observation of ovary of *Macrobrachium nipponense* after injection with *Mn-CH7D* dsRNA. OC: oocyte; N: nucleus; CM: cytoplasmic membrane; Scale bars: 400×.

On the last day of the experiment, tissue slices were taken from the ovaries of the shrimp. The results showed that the cell structure was unchanged, the nucleus was clearly visible, and there were no defects in the ovarian structure of the experimental and the female control shrimp (Figure 9).

## 3. Discussion

Ecdysteroids regulate many aspects of the developmental process and reproductive activity in insects and crustaceans [25,26,27]. Cholesterol and other sterols are indispensable precursors for the biosynthesis of steroid hormones, and the conversion of cholesterol to the next specific intermediate is an essential biochemical step across species [28,29]. In this study, we identified the Cholesterol 7-desaturase gene, a key gene in the first step of ecdysone synthesis, from the hepatopancreas transcriptome of *M.nipponense* and predicted that its coding region encodes 291 amino acids, including two conserved domains: the Rieske [2Fe-2S] domain and non-heme iron binding domain. The Rieske [2Fe-2S] domain and non-heme iron binding domain are involved in electron transfer and are essential for catalytic enzyme function [30]. It is, therefore, likely that *Mn-CH7D* itself has an enzymatic activity on sterols. *Mn-CH7D* can convert cholesterol into 7-dehydrogenated cholesterol, and many isoenzymes exist in insects and crustaceans, such as *DAF-36* [31] and *NVD* [32,33,34]. The mechanism of action is that cholesterol desaturase is used to convert cholesterol into 7-dehydrogenated cholesterol, and then through the action of a series of P450 enzymes, the active ecdysone with various structures is formed to act on target tissues and target cells, thus regulating and controlling the growth, deformation, molting, and development of insects and crustaceans. The phylogenetic tree showed that *Mn-CH7D* gene clustered into one branch in crustaceans and insects, indicating that *Mn-CH7D* is more conserved in the same class.

Tissue-specific analysis revealed that transcripts were detected at much higher levels in the ovaries of female adults than in other tissues, suggesting that the ovary of *M. nipponense* actively synthesized ecdysteroids, consistent with the previous reports [35]. The expression of *Mn-CH7D* peaked at O-III, which verified that *Mn-CH7D* was closely related to ovarian maturation [36]. Previous studies have shown that this period is the premolting stage of *M. nipponense*, suggesting that it is likely involved in the regulation of molting [37]. qPCR at different stages of the embryo showed that the expression of *Mn-CH7D* increased sharply from the nauplius stage (NS) to the zoea stage (ZS) and peaked at ZS. At the zoea stage, the embryonic development of *M. nipponense* was basically completed [38], indicating that *Mn-CH7D* played a critical role in hatching. Similar results were reported in the study of *Penaeoidean shrimp Sicyonia ingentis* by Chang E S et al., which found that in *Penaeoidean shrimp*, the content of molting steroid is very little after laying eggs, and it begins to increase in the embryonic development process. And it reached the maximum in the pre-hatching stage [39]. After membrane emergence, the expression level of *Mn-CH7D* began to increase until L10, and the expression level of L10 was the highest in the whole process of larva development, indicating that the function of *Mn-CH7D* gene is closely related to ontogeny, especially larva development. After metamorphosis, the expression level of *Mn-CH7D* reached the highest on the 15th day after metamorphosis (PL15), and there was no significant difference in other stages. At PL15, the gonads began to differentiate, suggesting that *Mn-CH7D* was associated with gonad development.

To further understand the biological roles of *Mn-CH7D* in reproductive processes, we knocked down *Mn-CH7D* using RNAi. The expression of *Mn-CH7D* in the ovaries of *M. nipponense* was significantly reduced by injection of dsRNA on day 4 (Figure 6). After silencing of *Mn-CH7D* resulted in the suppression of gonadal development. Studies have found that ecdysteroids play an important role in *Vg* synthesis [40,41]. After *Mn-CH7D* silencing, ecdysteroid synthesis is blocked, so gonad development is inhibited. Similarly, the silencing of the *Nvd* gene, which was highly expressed in females, leads to failure of ovarian development and subsequent egg laying [34]. *Nvd* and *Mn-CH7D* are isoenzymes, so the results are reliable. During the RNAi experiment, the GSI was calculated to further demonstrate gonad maturation. On the 14th day, the GSI of the experiment group injected with *Mn-CH7D* dsRNA was significantly lower than that of the control group. By day 14, the ovarian maturation of the experimental group remained at O-II, whereas the ovarian maturation of the control group reached O-IV, and gonadal development was close to maturity. GSI indicates that injection of *Mn-CH7D* dsRNA can effectively inhibit ovarian maturation, confirming the important role of Cholesterol 7-desaturase in gonad maturation. In addition, the ovarian structure of the female shrimp injected with *dsCH7D* and *dsGFP* was free from defects through tissue sections, indicating that silencing this gene only delayed ovarian development. 

After RNAi, the molting frequency of the experimental group was significantly lower than that of the control group on days 16–20 (*p <* 0.05) (Figure 8). Thus, successfully silencing *Mn-CH7D* in *M. nipponense* could significantly inhibit the molting of *M. nipponense*. This finding is consistent with the observations reported by Sumiya E et al., knockdown of *nvd* significantly resulted in the arrest of molting and growth by RNAi [33]. After the successful knockdown of *Mn-CH7D* in *M. nipponense*, we measured the content of ecdysone using ELISA. We found that the level of ecdysone was significantly decreased compared with the control group on days 7, 14, and 20 (*p <* 0.05). The level of ecdysone tends to increase on day 7, as the second molt begins on day 14, and the level of ecdysone will increase before molting, which has been demonstrated by previous studies [42,43]. These results suggested that the knockdown of the *Mn-CH7D* gene inhibited the synthesis of ecdysone, thereby affecting the molting of *M. nipponense*. Cholesterol is catalyzed to the active ecdysone by a series of P450 enzymes. Similar results were observed that the knockout of *Spook* and *CYP302a1* could inhibit molting and reduce the content of ecdysone [44,45]. It has also been described in other arthropods, including *Bemisia tabaci*, *Bombyx mori,* and *Locusta migratoria* [46,47,48].

These results strongly suggest that the *Mn-CH7D* gene is an ecdysone synthesis pathway gene, which is involved in ecdysone synthesis and plays an important role in the molting and ovarian maturation of crustaceans. This study demonstrated for the first time that Cholesterol 7-desaturase involved in ecdysone synthesis act as gonadotropin in *M. nipponense*, providing a theoretical basis for the artificial control of ecdysone. At the same time, it also provides strong evidence that this ecdysone is a key hormone in controlling reproductive ecdysone. 

## 4. Materials and Methods

### 4.1. Experimental Prawns and Breeding Conditions

Healthy female *M. nipponense* (body weight 0.56 ± 0.13 g and body length 3.68 ± 0.22 cm) were obtained from Freshwater Fisheries Research Center, Chinese Academy of Fishery Sciences, Wuxi, Jiangsu Province, China. No endangered or protected species were involved in this experiment. All experimental protocols and methods were approved in September 2022 (Authorization no. 20220901002) by the Animal Care and Use Ethics Committee in the Freshwater Fisheries Research Center (Wuxi, China). The prawns were raised in an indoor circulating aquaculture system (tanks, 1 m × 0.8 m × 0.5 m) to acclimate for one week. During the culture period, the water temperature was maintained at about 27 ± 1 °C. *M. nipponense* were fed a daily ration of paludina in the morning and evening. Each feeding amount depended on the weather, feeding conditions, timely adjustment, and clean food scraps once a day ensured water quality.

### 4.2. Sample Collection

Tissues of the eyestalk, brain, heart, hepatopancreas, gills, muscles, and ovaries of female *M. nipponense* were gathered, frozen immediately in liquid nitrogen, and then stored at −80 °C until use. Individuals of *M. nipponense* at different developmental stages (including embryonic developmental stage and larval stage) were also dissected, frozen, and stored at −80 °C separately for future experiments. The specific classification of ovarian stages and different stages of the embryo is listed in Table 1 based on the criteria studies previously [49].

### 4.3. Gene Cloning and Sequence Analysis of Cholesterol 7-Desaturase

The total RNA of *M. nipponense* at different developmental stages and different tissues was extracted using RNAiso Plus reagents (Takara, Japan) according to the manufacturer’s instructions, and quality was assessed by 1% agarose gel electrophoresis. The first-strand cDNA was synthesized with RNA as a template using the M-MLV reverse transcriptase (Takara, Japan) kit at 42 °C for 2 min. The reaction was finished after 37 °C for 5 min and 85 °C for 30 s. Then, the synthesized cDNA was kept at −20 °C for subsequent quantitative real-time PCR (qPCR) reaction to detect the expression pattern of *the Mn-CH7D* gene in *M. nipponense.* EIF was used as the internal reference gene [50]. The content of *Mn-CH7D* mRNA was calculated by the 2^−△△CT^ method [51].

The full-length DNA sequence of Cholesterol 7-desaturase was cloned using a 3′RACE kit according to the manufacturer’s instructions [52]. The full-length *Mn-CH7D* cDNA sequence determined by combining a subset of sequences from the *M. nipponense* hepatopancreas transcriptome library with the 3′race production was obtained. According to the Cholesterol 7-desaturase sequence, the primers used were designed by a prime designing tool (https://www.ncbi.nlm.nih.gov/tools/primer-blast/, accessed on 1 May 2022) (Table 2).

Domains in the protein sequence were determined via Conserved Domain Searches at NCBI (https://www.ncbi.nlm.nih.gov/Structure/cdd/wrpsb.cgi, accessed on 5 May 2022); amino acid sequence alignment was analyzed by DNAMAN 6.0 software; the open reading frame (ORF) of CH7D was predicted by the ORF Finder program (http://ncbi.nlm.nih. gov/gorf/gorf.html, accessed on 5 May 2022); signal peptide was predicted using Signal 5.0 Server (http://www.cbs.dtu.dk/services/Signal/P, accessed on 1 August 2022). Molecular weight (MW), the isoelectric point (PI), and amino acid composition were performed using ProtParam on the ExPASy website (https://web.expasy.org/protparam/, accessed on 1 August 2022). The phylogenetic tree was constructed based on the Cholesterol 7-desaturase protein and its orthologs in other species by the neighbor-joining (NJ) method using the MEGA 7.0 software.

### 4.4. In Situ Hybridization

Ovarian (stage I–V) samples fixed in 4% paraformaldehyde solution were used for in situ hybridization study. Based on the cDNA sequence of *Mn-CH7D*, the antisense and sense probes of Chromogenic in situ hybridization (ISH) with DIG signal were designed by Primer5 software and synthesized by Shanghai Sangon Biotech Company. More details of in situ hybridization have been described in previous studies [53]. The probe sequence of Mn-CH7D is as follows: 5′-CGGACCCTTTGAAGATGTTGGGACGATGGAG. The negative control uses the antisense chain of the sequence as the probe. The sequence is as follows: 5′-CTCCATCGTCCCAACATCTTCAAAGGGTCCG. HE represents the blank control groups with routine hematoxylin-eosin staining, and negative indicates the control groups poured with antisense probes. Positive suggests the experimental group with sense probes poured.

### 4.5. RNAi Experiment

#### 4.5.1. Interference Efficiency Detection

The primers used to synthesize double-stranded RNA (dsRNA) were designed with the online software Snap Dragon (https://www.flyrnai.org/cgi-bin/RNAi_find_primers.pl, accessed on 1 May 2022). The dsRNA of *Mn-CH7D* was synthesized using Transcript Aid™ T7 High Yield Transcription kit (Fermentas, Inc., Waltham, MA, USA), and its concentration was measured/determined at 260 nm using a BioPhotometer (Eppendorf, Hamburg, Germany). Expression levels of *Mn-CH7D* were measured by qPCR to determine the interference efficiency. 

A total of 300 healthy female prawns in stage IV were randomly distributed into 6 aquaculture tanks in equal proportion, which were the experimental group and the control group. The experimental group was injected with *Mn-CH7D* ds RNA at a dose of 4 μg/g (calculated per gram of body weight) through the pericardial cavity of *M. nipponense* [54], while the control group was injected with the same volume of *dsGFP*. The frequency of injections was five days. The *Mn-CH7D* expression of the ovary was investigated to detect the interference efficiency by qPCR.

#### 4.5.2. Ovarian Development and GONAD Somatic Index

The ovarian development was observed and recorded daily. The specific classification criteria are shown in Table 1. Three prawns were randomly selected from experimental and control groups on the 1st, 4th, 7th, 14th, and 20th day after injection, with 3 replicates per group. Among them, three shrimp were calculated for the gonad-somatic index (GSI). The prawns were weighed before and after the dissection of the ovaries. The GSI was calculated using the following formula: GSI = gonadal weight/body weight × 100%, as described previously [55].

#### 4.5.3. Molting Frequency and Ecdysis Hormone Content

The number of molts was observed and recorded daily. Molting frequency is calculated using the following formula: Molting frequency = (Nm/Ns)/D, where Nm is the total number of molts, Ns is the number of prawns in the aquarium, and D is the number of experimental days.

Three prawns were randomly selected from experimental and control groups on the 1st, 7th, 14th, and 20th day after injection, respectively. These shrimp were tested for hormone contents. The contents of ecdysone in prawns were determined by enzyme-linked immunosorbent assay with the double antibody two-step sandwich method, according to the Shrimp EHELISA Kit instruction. Experiments were performed in triplicate.

### 4.6. Tissue Section

At the end of the experiment, the ovaries of two shrimp from the experimental group and the control group were collected and fixed with 4% paraformaldehyde solution for tissue section preparation. The tissue slides were deparaffinized and hydrated using standard procedures and stained with hematoxylin and eosin (H&E). The final observation is a cross-section of the tissue.

### 4.7. Data Analysis

All quantitative data were presented as mean ± standard deviation (Mean ± SD). SPSS Statistics 24.0 was used for data analysis of the experimental data. The significant differences between the control group and the treatment group were analyzed by one-way ANOVA and Duncan test for multiple comparisons. The significance level of data variance was set at 0.05. The relative mRNA expression levels of the genes were calculated according to the 2^−ΔΔCt^ comparative CT method.

## 5. Conclusions

In this study, the full-length cDNA sequence of Cholesterol 7-desaturase from *M. nipponense* was successfully cloned and analyzed. The expression characteristics of Cholesterol 7-desaturase in different tissues and different stages of ovary and embryo development of *M. nipponense* were investigated. Importantly, this study demonstrated for the first time that this gene involved in ecdysone synthesis act as gonadotropin in *M. nipponense*, providing a theoretical basis for the artificial control of ecdysone. It also provides some new insights and ideas to solve the production problem of rapid sexual maturation, which is of great significance to the development of the crustacean industry.

## Figures and Tables

**Table 1:** Different developmental stages of ovary and embryo of *M. nipponense*.

Stages	Characteristic
O-I	undeveloped stage, transparent
O-II	developing stage, yellow
O-III	nearly-ripe stage, light green
O-IV	ripe stage, dark green
O-V	worn out stage, gray
CS	cleavage stage
BS	blastula stage
GS	gastrulation stage
NS	nauplius stage
ZS	zoea stage
L1	the 1st-day larvae after hatching
L5	the 5th-day larvae after hatching
L10	the 10th-day larvae after hatching
L15	the 15th-day larvae after hatching
PL1	the 1st day after metamorphosis
PL5	the 5th day after metamorphosis
PL10	the 10th day after metamorphosis
PL15	the 15th day after metamorphosis
PL20	the 20th day after metamorphosis
PL25	the 25th day after metamorphosis

**Table 2:** Primers for cDNA clone, qPCR analysis, and RNAi involved in this study.

Primer’s Name	Sequence (5′-3′)	Usage
CH7D F1	CTAATTGCGCTAAAGTCCCGAAG	ORF
CH7D R1	GGTCATGGTGAGGATCTTGAACT	ORF
CH7D F2	TTGCAGACAATCAGGTTTTGGAC	ORF
CH7D R2	CTCACAGAGCAGGACGAATTTTG	ORF
CH7D F3	GTGCATCAGTTCTATTCGTCGTC	ORF
CH7D R3	CTGAGCTTTGTACTGCTTGTTGT	ORF
CH7D 3′F1	GCTGAGAGTAGAACTGCGCGTAC	3′RACE
CH7D 3′F2	TCTCGTTCCTGAAGGAGAACTGC	3′RACE
CH7D F	TGGAACAACAAGCAGTACAAAG	qPCR
CH7D R	CTGGCTGTTCTGAGAGTAGAACT	qPCR
EIF F	CATGGATGTACCTGTGGTGAAAC	qPCR
EIF R	CTGTCAGCAGAAGGTCCTCATTA	qPCR
CH7D dsF	TAATACGACTCACTATAGGGCGCTAAAGTCC CGAAGACAG	dsRNA
CH7D dsR	TAATACGACTCACTATAGGGACGAATTTTGC GTAAGGTGC	dsRNA
GFP dsF	TAATACGACTCACTATAGGGACGAAGACCTT GCTTCTGAAG	dsRNA
GFP dsR	TAATACGACTCACTATAGGGAAAGGGCAGAT TGTGTGGAC	dsRNA

## Data Availability

The data presented in this study are available upon request from the corresponding author.
